# Splenectomy is associated with sexual dysfunctions and decreased libido

**DOI:** 10.1038/s41598-021-01371-7

**Published:** 2021-11-05

**Authors:** Kelly Renata Sabino, Andy Petroianu

**Affiliations:** Department of Surgery, School of Medicine, Federal University of Minas Gerais, Avenida Afonso Pena 1626 - apto. 1901, Belo Horizonte, 30130-005 Brazil

**Keywords:** Immunology, Neuroscience, Physiology, Structural biology, Environmental sciences, Environmental social sciences, Diseases, Gastroenterology, Health care, Medical research, Signs and symptoms, Urology

## Abstract

The removal of the spleen due to disease or trauma may be followed by disorders due to the asplenism, including immunodeficiency, hematological and metabolic diseases, mainly dyslipidemia, which can lead to sepsis, pulmonary embolism and early death. Although patients frequently report sexual disinterest after splenectomy, this feature has been experimentally studied only in a translational investigation performed by the same group of this work. To study libido and other sexual functions after the complete removal of the spleen in humans. This study was performed on 60 healthy adults, 30 men and 30 women, after more than 1 year of total splenectomy to treat isolated splenic trauma. The International Index of Erectile Function was applied to men and the Female Sexual Function Index to women. The analysis compared the responses obtained in the periods before and after the splenectomy. Laboratory tests with hematological and biochemical analyses, including sex hormones, were performed in all patients. The pre- and postoperative results were compared in each group using the paired t-test, with each patient being his or her own control and with significance to p < 0.05. A decrease in libido and an increase in sexual dysfunction was observed after splenectomy in all men and women, p < 0.001. All postoperative laboratory tests showed normal values in both genders. Asplenia is associated with a marked decrease in libido and intense sexual dysfunction in both men and women, with normal hematological and biochemical laboratory tests, including hormonal exams.

## Introduction

During the Battle of Dettingen in England (1743), a soldier suffered abdominal trauma with externalization of the spleen, which was surgically removed. He recovered from the surgical procedure, but lost interest in sexual activity^1^. Shulz and Czermak, in the nineteenth century, observed a reduction in fertility in splenectomized animals. These two publications indicated the possibility that splenectomy could interfere in sexual activity^1,2^. In personal clinical experience with splenectomized patients, it was found that, after the removal of the spleen, many patients of both genders complained about the loss of sexual interest. The removal of the spleen due to disease or trauma may lead to adversities resulting from the asplenic state, including immune, hematological and metabolic disorders, mainly dyslipidemia, early death due to sepsis and pulmonary embolism^3–9^. The only published study that relates sexual activity to the spleen or splenectomy belongs to the authors of this study^9^. The previous work, carried out on couples of mice, started this translational investigation and found a reduction in the number of pregnancies after splenectomy. Another finding of that study was the reduction in the number of puppies from couples who had a pregnancy after splenectomy^9^. The purpose of this study was to verify changes in libido and other sexual functions after the complete removal of the spleen.

## Methods

This study belongs to a line of research and was carried out in accordance with the recommendations of the Declaration of Helsinki and was approved by the Research Ethics Committee of the Hospital Foundation of the State of Minas Gerais, logged under protocol number 068934/2016. All patients agreed to participate in the study, and signed the Free and Informed Consent.

Medical records of patients of both genders, between 20 and 50 years of age, with no selection between men and women, who underwent a total splenectomy due exclusively to splenic blunt trauma, without other diseases, at the João XXIII Emergency Hospital in Belo Horizonte, Brazil, between 2010 and 2018, were collected, before the COVID-19 pandemic. All patients corresponding to these records were invited to a medical consultation, where this project was presented and explained to them. This study included only patients who agreed to participate in this research and who had an active sexual relationship before splenectomy, presenting no diseases, such as diabetes mellitus, vasculopathies, heart disease, malignant neoplasms, mental disorders, or addictions of smoking, alcoholism, the use of psychotropics, etc. Patients whose answers to the questionnaires were doubtful or incomplete, as well as those who did not perform all the complementary exams, were excluded from this study and replaced by other patients in similar conditions. At the end of this selection, 30 men and 30 women were studied with all the data collected reliably only by the authors of this work.

Each patient underwent a careful anamnesis about their health, including immunological manifestations, current physical condition and complaints, as well as previous illnesses. Then all of them underwent a complete physical examination. After that, the questionnaires were answered in full by the 60 patients, who, at the time of their splenectomy, were between 20 and 44 (M = 30) years of age for men and between 19 and 47 (M = 34) years of age for women. Regarding marital status, in the male gender, eleven declared themselves to be single, eight, in a stable relationship, six married, and five, divorced. In the female sex, seven declared themselves to be single, nine, in a stable relationship, eight married, and six, divorced.

Each patient was identified by age and post-splenectomy time. The International Index of Erectile Function (IIEF) was then applied to men and the Female Sexual Function Index (FSFI) to women^10–14^. For each question in the questionnaire, two responses were collected for the periods before and after the splenectomy. The postoperative study was conducted over a period of one to 9 (5 ± 3) years from the splenectomy.

Patients underwent blood count on Sysmex XN-3000 hematology analyser (Sysmex America, IL-USA). The hematological tests included thrombocytes, erythrocyte characteristics, Howell Jolly, Heintz and Pappenheimer bodies. The leukocytes were classified by either their structure and by cell lineage, including B and T lymphocytes, separately studied. Creatinine, glucose, lipidogram, fractionated proteins, aminotransferases, luteinizing hormone (LH), follicle stimulating hormone (FSH), thyroid stimulating hormone (TSH), free thyroxine (T4), free testosterone, prolactin, estradiol, cortisol, adrenocorticotropic hormone (ACTH) and dehydroepiandrosterone were assessed using an Ortho VITROS^®^ 5600 chemiluminescence immunoassay (CLIA) analyzer (Ortho Clinical Diagnostics, Skillman, NJ-USA) for detection of biochemical immunological and hormonal exams.

The interpretation of the IIEF questionnaire for males followed the guidelines in Table 1. Each domain consisted of three to six objective questions, restricted to their specific aspects.

**Table 1:** Classification of male sexual function through the domains of the international index of erectile function and values assigned to each domain by this index.

Domain	Severe dysfunction	Moderate dysfunction	Without dysfunction
Erectil function (Q1)	5–10	11–24	25–30
Orgasm (Q2)	2–4	5–8	9–10
Sexual desire (Q3)	2–4	5–8	9–10
Sexual satisfaction (Q4)	3–5	6–12	13–15
General satisfaction (Q5)	2–4	5–8	9–10

The interpretation of the FSFI questionnaire for women followed the guidelines in Table 2. Each domain was made up of two to four objective questions, restricted to their specific aspects.

**Table 2:** Classification of female sexual function through the domains of the Female Sexual Function Index and values assigned to each domain by this index.

Domain	Severe dysfunction	Moderate dysfunction	Without dysfunction
Sexual desire (Q1)	1.2–2.4	3.0–4.8	5.4–6.0
Sexual excitement (Q2)	1.2–2.1	2.4–4.8	5.1–6.0
Vaginal lubrication (Q3)	1.2–2.1	2.4–4.8	5.1–6.0
Orgasm (Q4)	1.2–2.0	2.4–4.8	5.2–6.0
Sexual satisfaction (Q5)	1.2–2.0	2.4–4.8	5.2–6.0
Pain during sexual activity (Q6)	1.2–2.0	2.4–4.8	5.2–6.0

In addition to these questionnaires, each patient was asked about the number of children they had after splenectomy.

The results were calculated for means and standard deviation of means (SDM), in addition to percentages, as measures to describe the parameter incidence. When comparing the measurements made on the same patient before and after splenectomy, the paired t-test was used to determine the difference between two measurements taken on the same patient. All results were considered significant for a probability of greater than 95%, corresponding to p < 0.05.

## Results

All patients reported having had an active sex life until abdominal trauma, which only injured the spleen. All patients underwent laparotomy and removal of the spleen exclusively, with no need for further surgical procedures. The patients had no postoperative complications or other conditions related to splenectomy, no disease was found, and no patient was using medication or psychotropic drugs. No patient presented any symptom related to immunological deficiency after splenectomy, including pneumonia, urinary or genital infection, sepsis, meningitis and frequent influenza episodes.

In males, the mean erectile function score decreased after splenectomy, indicating a worsening libido and the appearance of sexual dysfunctions. There was a reduction in all studied domains, with a greater worsening of sexual desire (Table 3).

**Table 3:** Comparative analysis of male sexual function scores (mean ± standard deviation of mean) of the international index of erectile function domains.

Parameter	Preoperative	Postoperative	P
Erectile function (Q1)	4.74 ± 0.31	2.49 ± 1.39	0.001
Orgasm (Q2)	4.82 ± 0.23	2.33 ± 1.66	0.001
Sexual desire (Q3)	4.57 ± 0.43	1.87 ± 1.16	0.001
Sexual satisfaction (Q4)	4.81 ± 0.23	2.48 ± 1.06	0.001
General satisfaction (Q5)	4.85 ± 0.24	2.48 ± 0.95	0.001

*P* significance related to paired t-test.

When asked about erectile function, 100% of patients reported having been normal in the preoperative period and that erectile dysfunction occurred only after undergoing the splenectomy, which was reported by 80% of the patients. Ejaculatory and orgasm dysfunction also appeared in 80% of the patients after undergoing the splenectomy. Preoperatively, only four (13.3%) patients reported having moderate orgasm and ejaculation dysfunction.

Sexual desire was totally lost by 70% of the men after undergoing the splenectomy, and only one patient reported maintaining normal, unchanged erectile function after the procedure. Before the splenectomy, 86.7% of the patients reported having a normal sexual desire, only 13.3% of the patients had sexual dysfunction, which, according to their reports, was not intense. In the preoperative period, sexual intercourse was reported to be satisfactory for all patients; however, there was a decrease in sexual satisfaction after undergoing a splenectomy in 40% of them. When asked about general satisfaction regarding preoperative sexual function, only one (3.3%) patient reported moderate complaints, but after having undergone the splenectomy, the patient reported moderate and severe dissatisfaction in sexual activity in 96.7% of the patients.

In females, a reduction was found in the score of all studied domains, when the periods before and after splenectomy were compared (Table 4). Satisfaction in sexual activity was the domain with the highest drop in scores, followed by reductions in orgasm and sexual desire. In the preoperative period, the domain that had the lowest average score was the natural vaginal lubrication, which was reported as reduced and unsatisfactory, while in the postoperative period, the lowest average score was the decrease in sexual desire. Reduction or loss of sexual desire occurred in all patients after undergoing the splenectomy, whereas in the preoperative period, this disorder was present in 43.4% of them.

**Table 4:** Comparative analysis of female sexual function scores (mean ± standard deviation of mean) of the Female Sexual Function Index domains, before and after splenectomy.

Domains	Preoperative	Post operative	P
Sexual desire (Q1)	5.00 ± 0.82	2.76 ± 1.05	0.001
Sexual excitement (Q2)	5.01 ± 0.34	2.63 ± 1.35	0.001
Vaginal lubrication (Q3)	4.96 ± 0.50	3.12 ± 1.40	0.007
Orgasm (Q4)	5.46 ± 0.59	2.74 ± 0.65	0.001
Sexual satisfaction (Q5)	5.49 ± 0.54	2.58 ± 0.83	0.001
Pain during sexual activity (Q6)	5.78 ± 0.61	3.74 ± 1.33	0.001

*P* significance related to paired t-test.

Asked about arousal for sexual intercourse, 56.6% of the patients revealed severe dysfunction after undergoing a splenectomy, while in only 6% of them this function was unchanged. Most patients (93.3%) felt a decline in the natural vaginal lubrication, and 100% sexual dissatisfaction after undergoing a splenectomy. There was an increase in complaints of pain related to sexual intercourse from 13.3% before surgery to 86.7% after the splenectomy.

None of the 60 patients had children after the splenectomy. However, it should be considered that 12 (40%) men, and 8 (26.6%) women underwent splenectomy less than three years before this study and about half of the patients were not married or in a stable relationship.

All laboratory test results were within normal reference values. No abnormal event was found in the hematological study, all elements were normal and no atypical cytopathological body was found. Only free testosterone values were close to the lower limit of normal in 90% of the men. However, the averages of the sexual function domains of patients who had normal testosterone and those with low values did not differ (Table 5).

**Table 5:** Comparative analysis of female and male hormone levels (mean ± standard deviation of mean) after splenectomy.

Exams	Female	Male
LH	22.89 ± 13.71 mUI/ml	1.22 ± 0.83 mUI/ml
FSH	63.23 ± 21.59 mUI/ml	4.71 ± 3.31 mUI/ml
Progesterone	41.11 ± 2.38%	0.43 ± 0.33 ng/ml
17αhidroxiprogesterone	1.41 ± 1.22 ng/dl	0.98 ± 0.76 ng/dl
Estradiol	11.32 ± 45.7 pg/ml	23.16 ± 0.64 pg/ml
Androsterone (SDHEA)	97.00 ± 53.21 mcg/dl	190.83 ± 7.85 mcg/dl
Testosterone (free)	1.20 ± 0.33 pg/mla	6.57 ± 1.58 pg/ml
Prolactine	12.70 ± 5.10 ng/dl	13.5 ± 4.3 nd/dl
Cortisol (basal)	12.70 ± 3.72 µg/dl	16.77 ± 5.99 µg/dl
TSH	2.33 ± 1.29 µg/dl	2.57 ± 1.1 mUI/l
T4 (free)	1.51 ± 0.27 ng/l	1.45 ± 0.21 ng/l
ACTH	28.25 ± 14.60 pg/ml	31.44 ± 12.3 pg/ml

*LH* luteinizing hormone, *FSH* follicle stimulating hormone, *TSH* thyroid stimulating hormone, *T4* thyroxine, *ACTH* adrenocorticotropic hormone and dehydroepiandrosterone.

## Discussion

Both male and female sexual functions are associated with multiple organic and psychosocial factors. To reduce the intervening factors, this series was restricted to healthy patients, of reproductive age, and with active sexual activity, without the illegal use of drugs or psychotropic drugs, smoking, or alcoholism^1,3–6^. Surgical trauma was restricted to the spleen, which was the only injured organ in all cases and must be fully removed. Thus, sexual dysfunctions that arose after splenectomy could be associated with asplenic status. The assessment of the sexual functions used internationally accepted accurate questionnaires^10–14^. According to the literature, sexual dysfunctions are more frequent in women (43%) than in men (31%)^15^. In females, hypoactive sexual desire is the most common dysfunction (27%), followed by dyspareunia (23%) and the absence of a satisfactory orgasm (21%)^16^. In this study, the dysfunction most reported by patients in the preoperative period was low sexual excitement (53.3%), followed by hypoactive sexual desire (43%), and little vaginal lubrication (30%). In the postoperative period, all patients had more than one manifestation of sexual dysfunction (Table 4). Despite the extensive literature review, no other surgical procedure was found that resulted in sexual dysfunction in all patients.

Salto et al. found delayed ovulation and a higher incidence of pseudopregnancy in asplenic rats. These disorders were normalized after the injection of splenocytes, indicating an association of splenic tissue with ovarian functions^17^. Okely et al. studied the dynamics of ovulation associated with mediators of acute inflammation and observed fewer leukocytes in the ovaries of splenectomized rats, indicating that the spleen releases leukocytes for the defense of the ovary in the ovulatory period^18^. No other studies were found that related the spleen to organs of the reproductive system except a recent article published by the authors of this work^9^. In men, sexual dysfunctions are well-known and estimated in 30% of adults, with a predominance of uncontrolled erection and ejaculation^15,19^. In this study, the prevalence of sexual dysfunction before splenectomy was 15%, which proved to be lower than that described in the literature. The main sexual dysfunctions were also different, with a prevalence of underactive sexual desire and difficulties in reaching an orgasm. After splenectomy, 96.7% of the men had more than one manifestation of sexual dysfunction. This specific characteristic of the asplenic state has not been previously reported and similar sexual disorders were not found in other conditions, considering that all men were normal, without disease or using any agent that interferes in sexual functions.

It was long believed that the spleen was a superfluous organ and that its removal did not threaten the patients’ health. However, recent studies have shown the multiplicity of functions that the spleen has and the great risks of the asplenic state^3–8^. The spleen is an essential organ of the hematological system since the embryonic phase, and it is the main responsible for the acute defense against the invasion of microorganisms, including bacteria, fungi and viruses^1,3,4^. It is also related to lipid metabolism and is responsible for part of the functions attributed to the liver, such as the production of bilirubins and amino acids^20–22^. On the other hand, the asplenic state is related to a high incidence of sepsis, pulmonary embolism, increased peripheral insulin resistance and dyslipidemia, associated with early death^1,3,4,23^. In order to determine the link between splenectomy and sexual dysfunction, it was necessary not only to conduct the two questionnaires, but also to study changes in the hematological characteristics, hormonal profile and immunological status Despite the risks of the asplenic state, none of the patients in this work and following the studied parameters presented clinical or laboratory hematological, immune, endocrine or metabolic disorders after the removal of the spleen. The presence of cytopathological bodies, such as Howell Jolly, Heintz and Pappenheimer may be found in the asplenic state, but none of these atypical cells was found in this study. However, it must be emphasized that none of these patients was old or presented any disease, and all the data were collected until 2019 before the COVID-19 pandemic. According to the care clinical assessment, none of patients presented any vascular or neurologic disorder, which could be related to sexual dysfunction. Due to the complete absence of clinical abnormality, no functional test of peripheral vascular or nervous system was justified.

The association between splenomegaly and delayed somatic and sexual development in children and teenagers is well known^24^. Nonetheless, its etiopathogenesis is still unknown and the only effective treatments are total or subtotal splenectomies, which completely reverse this hypodevelopment in a short time, as long as performed before the consolidation of bone epiphyses^24,25^. In the experience of the authors of this work, partial preservation of the spleen prevents complications from the asplenic state without compromising the normal somatic and sexual development^25^. This work evidenced the worsening of sexual functions in both men and women, according to a previous study carried out following the same line of this translational investigation^9^. In couples of mice, there was not only a reduction in the frequency of pregnancies after splenectomy, but also a lower number of pups in each pregnancy^9^. This is the only work that relates the spleen to sexual and reproductive activities, and it should be noted that, as previously verified experimentally, in this clinical study, there was also no pregnancy after the splenectomy. Considering the results of this previous experimental study, to verify the spermatozoa of the male patients in this work seemed logical and, depending on results, important informations could be associated with sexual behavior disorders. However, most of men did not agree to collect their sperm. It must be highlighting once more that all patients presented sexual dysfunction and reduction of their libido.

The spleen is pivotal in the immune and reticuloendothelial systems, and it is associated with coagulation cascades. Asplenic patients are at risk for several complications, including overwhelming sepsis and vascular thrombosis associated with intimal fibrosis and plexiform lesions such as seen in pulmonary thromboembolism that may occur in 30% of patients^3,6,26–29^. These adverse effects of splenectomy are mainly found in elderly and hematologic diseases, mainly thrombocytopenia and myelofibrosis^1^. According to the literature review, none of these complications was linked to sexual dysfunction in both genders. Specifically, erectile dysfunction has been associated with vascular disorders such as microembolism, stenosis and dyslipidemia, which may occur after splenectomy^4,7,27–29^. However, according to the patients of this study the erectile dysfunction was not due to erectile physical difficulty or inability, but to sexual indifference or disinterest.

In the surgical experience, changes in behavior have been observed after the removal of several organs, such as lung, stomach, colon, uterus, etc. This behavioral change is attributed to operative trauma and organic complications resulting from the absence of the removed organs. However, it is necessary to consider that all organs have a large number of neurons and endocrine cells capable of secreting multiple mediators that act in several well-established organic functions. It is worthwhile to investigate whether there is a relationship between organs other than the brain in human behavior and that of other animals through neuroimmunoendocrine mechanisms. In this sense, the spleen most likely interferes in sexual behavior and the reproductive system.

## Conclusion

Asplenia is associated with a marked decrease in libido and intense sexual dysfunction in both men and women, with normal hematological and biochemical laboratory tests, including hormonal exams.

## Data Availability

All data underlying this article are available and will be immediately shared on reasonable request to the corresponding author*.*
